# Pattern-Based Phylogenetic Distance Estimation and Tree Reconstruction

**Published:** 2007-02-25

**Authors:** Michael Höhl, Isidore Rigoutsos, Mark A. Ragan

**Affiliations:** 1 Institute for Molecular Bioscience, The University of Queensland, Brisbane QLD 4072, Australia; 2 Australian Research Council Centre in Bioinformatics; 3 Bioinformatics and Pattern Discovery Group, IBM Thomas J Watson Research Center, Yorktown Heights, NY 10598, U.S.A

**Keywords:** alignment-free methods, phylogenetics, distance estimation, pattern discovery

## Abstract

We have developed an alignment-free method that calculates phylogenetic distances using a maximum-likelihood approach for a model of sequence change on patterns that are discovered in unaligned sequences. To evaluate the phylogenetic accuracy of our method, and to conduct a comprehensive comparison of existing alignment-free methods (freely available as Python package decaf + py at http://www.bioinformatics.org.au), we have created a data set of reference trees covering a wide range of phylogenetic distances. Amino acid sequences were evolved along the trees and input to the tested methods; from their calculated distances we infered trees whose topologies we compared to the reference trees.

We find our pattern-based method statistically superior to all other tested alignment-free methods. We also demonstrate the general advantage of alignment-free methods over an approach based on automated alignments when sequences violate the assumption of collinearity. Similarly, we compare methods on empirical data from an existing alignment benchmark set that we used to derive reference distances and trees. Our pattern-based approach yields distances that show a linear relationship to reference distances over a substantially longer range than other alignment-free methods. The pattern-based approach outperforms alignment-free methods and its phylogenetic accuracy is statistically indistinguishable from alignment-based distances.

## 1. Introduction

Tasks like database searching, sequence classification, phylogenetic tree reconstruction and detection of regulatory sequences are ubiquitous in bioinformatics. Most methods performing these tasks are based on (automated) alignments; however, alignment-free methods exist for solving the tasks. Recent years have seen an increasing number of alignment-free methods (reviewed in Vinga and Almeida 2003; see also Snel et al 2005; Vinga et al 2004; Van Helden 2004; Pham and Zuegg 2004; Burstein et al 2005; Wu et al 2005). In contrast to methods based on (automated) alignments, alignment-free methods make fewer assumptions about the nature of the sequences they work on, and so far are mostly devoid of any evolutionary model of sequence change (the only exception being the W-metric by Vinga et al 2004). The absence of an assumption of collinearity over long stretches (implicit in any alignment) destines them to be especially useful for handling DNA sequences that have undergone recombination, proteins with shuffled domains, and genomic sequences (which often feature large-scale rearrangements).

Previously, several alignment-free methods have been compared systematically for classification purposes and their ability to detect regulatory sequences. However, surprisingly little is known about their accuracy in phylogeny reconstruction. So far, new methods have been verified on a few trees only. A systematic study is sorely lacking in this field of research.

In Section 2 below we describe several alignment-free methods that we included in our comparison. We propose a new alignment-free method based on patterns in sequences, and a variant thereof, in Section 3. The phylogenetic accuracy of these methods is comparatively evaluated on synthetic and empirical data, covering a wide range of phylogenetic distances, and we assess whether differences are statistically significant in Section 4 before presenting conclusions.

## 2. Previous work

In this section, we provide a summary of previously established alignment-free methods. The first two were reviewed in Vinga and Almeida (2003), while the remaining six methods are more recent and are compared here for the first time. We discuss the following: *d^E^*, the (squared) Euclidean distance; *d^S^*, the standardized Euclidean distance; *d^F^*, a distance based on the fractional common *k*-mer count; *d^P^*, a distance based on probabilities of common *k*-mer counts under a multiplicative Poisson model; *d^C^*, the composition distance; *d^W^*, the W-metric; *d^LZ^*, a distance based on Lempel-Ziv complexity; *d^ACS^*, a distance based on the Average Common Substring length. Methods *d^S^* and *d^P^* require a set of equilibrium frequencies of amino acids. We supply amino acid frequencies used in the derivation of the JTT model (Jones et al 1992).

We represent a biological sequence by a string *X* (*Y*) of *n* (*m*) characters taken from the alphabet 𝒜 which contains *c* different characters {*a*_1_,…, *a_c_*}, eg all amino acids. Most alignment-free methods operate on words of length *k*, so-called *k-*mers: there are *w* = *c^k^* such different words. We represent the set of *k*-mers in *X* (or a derived property) by vector **v**^X^ = (*v*_1_*^X^*, …, *v_w_^X^*) (similarly for *k*-mers in *Y*); the parameter *k* is always implied. Each vector element describes the abundance of *k*-mer *i*.

The (squared) Euclidean distance was introduced into sequence comparison by Blaisdell (1986). The distance between *X* and *Y* is calculated using *c_i_^X^*(and *c_i_^Y^*), the count of *k*-mer occurrences in *X* (and *Y*).

(1) $$d^{E}(X,Y)=\sum_{i=1}^{w}{\left(c_{i}^{X}-c_{i}^{Y}\right)}^{2}$$

Later, Blaisdell (1989) found that *d^E^* yields values about twice the number of mismatch counts obtained from alignments.

The standardized Euclidean distance was found to improve on *d^E^* without incurring the computational problems associated with the slightly better performing Mahalanobis distance (Wu et al 1997).

(2) $$d^{S}(X,Y)=\sum_{i=1}^{w}{\left(f_{i}^{X}/s_{i}^{X}-f_{i}^{Y}/s_{i}^{Y}\right)}^{2}$$

Divide *f_i_^X^* (*f_i_^Y^*), the relative frequencies of *k*-mer occurrences in *X* (*Y*), by their standard deviations *s_i_^X^* (*s_i_^Y^*) as calculated from a set of equilibrium frequencies (Gentleman and Mullin 1989).

Edgar (2004a) described the fractional common *k*-mer count; it is used in a distance measure that speeds up guide tree construction in MUSCLE (Edgar 2004b).

Let *C_i_^XY^* = min (*c_i_^X^*, *c_i_^Y^*) denote the common *k*-mer count.

(3) $$F(X,Y)=\sum_{i=1}^{w}C_{i}^{XY}/[\text{min}\;(n,m)-k+1]$$

(4) $$d^{F}(X,Y)=-\text{log}\;(ɛ+F)$$

*F*, the fraction of common *k*-mers between *X* and *Y*, ranges from 0 to 1 and *d^F^* transforms this into a distance: *ɛ*, a small value added to prevent taking the logarithm of zero (at least in Edgar 2004a), is 0.1 there but 0.02 in MUSCLE. Both versions employ *d^F^* in slightly different ways; here, we directly use this common basis with *ɛ* = 0.1.

Van Helden (2004) compared several metrics for their suitability in classifying genes based on their regulatory sequences. He found a similarity measure based on probabilities from common *k*-mer counts under a multiplicative Poisson model to be best-performing. In our adaptation, we directly use the probabilities from Equation 5 without transforming them into similarities.

(5) $$P\left(x\geq C_{i}^{XY}\right)=\{\begin{array}{ll} {\left[1-F^{P}\left(C_{i}^{XY}-1,E_{i}\right)\right]}^{2} & \text{if }C_{i}^{XY}>0,\\ 1 & \text{else}. \end{array}$$

(6) $$d^{P}(X,Y)={\left[\prod_{i=1}^{w}P(x\geq C_{i}^{XY})\right]}^{1/w}$$

Here, in the calculation of *C_i_^XY^* the occurrence counts of *k*-mers are filtered to obtain recurrence counts, thereby justifying the Poisson assumption. If *k*-mer *i* occurs more than once in a sequence, we record the starting positions (*p*_1_, …) and proceed as follows. We accept *p*_1_ and discard all positions overlapping the corresponding *k*-mer. We repeat this process with the remaining positions until the list is exhausted; the accepted positions represent recurrences of *i. F^P^* refers to the Poisson probability distribution function and its parameter *E_i_* is the expected count under a set of equilibrium frequencies. *P*(*x* ≥ *C_i_^XY^*) is the probability that we observe a *k*-mer count at least as high as that between *X* and *Y*.

The last word-based alignment-free method considered here is the composition distance of Hao and Qi (2004). Under a Markov model for *k*-mer occurrence of order *k* − 2 (assuming word length *k* ≥ 3) we predict the probability *p*^0^ of a word (the *c_i_* refer to its characters) from the probabilities *p* (ie relative frequencies) of appropriate shorter subwords. To obtain the expected count *E^X^* of a *k*-mer in *X*, we re-arrange Equation 7 slightly.

(7) $$p^{0}(c_{1},\ldots ,c_{k})\equiv \frac{p(c_{1},\ldots ,c_{k-1})\;p(c_{2},\ldots ,c_{k})}{p(c_{2},\ldots ,c_{k-1})}$$

(8) $$\begin{array}{l} E^{X}(c_{1},\ldots ,c_{k})=\\ \frac{c^{X}(c_{1},\ldots ,c_{k-1})\;c^{X}(c_{2},\ldots ,c_{k})}{c^{X}(c_{2},\ldots ,c_{k-1})}\times \\ \frac{\left(n-k+1)\;(n-k+3\right)}{{\left(n-k+2\right)}^{2}} \end{array}$$

We can now assemble the composition vector (Hao and Qi 2004) for *k*-mer occurrence counts *c^X^* in *X*: **v***^X^* = (**c***^X^* − **E***^X^*)/**E***^X^*. Then we calculate the correlation between *X* and *Y* as the cosine of the angle between their composition vectors, and obtain a normalized dissimilarity *d^C^*.

(9) $$\text{cos}\;(X,Y)=\frac{\sum_{i=1}^{w}v_{i}^{X}v_{i}^{Y}}{{\left[\sum_{i=1}^{w}{\left(v_{i}^{X}\right)}^{2}\times \sum_{i=1}^{w}{\left(v_{i}^{Y}\right)}^{2}\right]}^{1/2}}$$

(10) $$d^{C}(X,Y)=\frac{1-\text{cos}\left(X,Y\right)}{2}$$

Vinga et al (2004) introduced the W-metric which they categorise as “word-based” but we note that for amino acid sequences (to which it was applied originally), it effectively operates on 1-mers only:

(11) $$\begin{array}{r} d^{W}(X,Y)=\sum_{i=1}^{w}\sum_{j=1}^{w}\left(f_{i}^{X}-f_{i}^{Y}\right)\\ \times \left(f_{j}^{X}-f_{j}^{Y}\right)\cdot W_{ij} \end{array}$$

Differences in amino acid composition, *f_i_^X^* − *f_i_^Y^*, between all pairs of amino acids, are weighted by their entries *W_ij_* in scoring matrix **W**. Vinga et al (2004) found their results virtually the same for different matrices (BLOSUM62, BLOSUM50, BLOSUM40 and PAM250); we use BLOSUM62 ( Henikoff and Henikoff 1992).

Otu and Sayood (2003) showed how Lempel-Ziv complexity, computed in a simple fashion utilizing two elementary operations (Lempel and Ziv 1976), can be used to define distance measures. We examine their final measure (that they call d_1_^**^): *c*(*X*) denotes the Lempel-Ziv complexity of *X*, and *XY* refers to the concatenation of *X* and *Y*.

(12) $$\begin{array}{l} d^{LZ}(X,Y)=\\ \frac{c(XY)-c(X)+c(YX)-c(Y)}{\frac{1}{2}[c(XY)+c(YX)]} \end{array}$$

Most recently, Burstein et al (2005) proposed the Average Common Substring (ACS) approach. They define *L*(*X, Y)* = ∑*_i_*_=1_*^n^* ℓ*_i_^XY^*/*n*, where ℓ*_i_^XY^* is the length of the longest string starting at *X_i_* that exactly matches a string starting at *Y_j_*. *L* provides a normalized length measure, from which we obtain an intermediate (asymmetric) distance *d* and finally *d^ACS^*.

(13) $$d\;(X,Y)=\frac{\text{log}\;(m)}{L\;(X,Y)}-\frac{\text{log}\;(n)}{L\;(X,X)}$$

(14) $$d^{ACS}(X,Y)=\frac{1}{2}[d(X,Y)+d(Y,X)]$$

## 3. Pattern-Based Approach

We use pattern discovery to find regions of similarity (presumed homology) occurring in two or more sequences; no alignment is necessary. To estimate phylogenetic distances, the patterns are considered to be local alignments. Adopting this point of view enables us to apply an established maximum-likelihood (ML) approach. Both the application of pattern discovery, and distance estimation by ML, represent novel steps in this context. We infer pairwise distances from the sequence data covered by patterns, yielding a distance matrix. Distance-based tree inference then proceeds by conventional means.

There is a fundamental distinction between our pattern-based approach and word-based methods: patterns are expressive enough to accommodate similar but non-identical stretches of sequence (see below), allowing us to calculate distances from the differences contained therein. Methods based on words cannot extract differences between two sequences from the sequence data covered by a *k*-mer that, by definition, occurs identically in these sequences.

### 3.1 Terminology

We briefly introduce some basic terminology for TEIRESIAS; for more details, including a description of the algorithm, the reader is referred to Rigoutsos and Floratos (1998). Let 𝒜 denote the alphabet of characters, eg all amino acids. Let ‘.’ ∉ 𝒜 be the wildcard character that represents any amino acid. Define a pattern *P* to be the regular expression 𝒜(𝒜 ∪ {‘.’})*𝒜, ie *P* starts and ends with amino acids, and may contain wildcard characters. A subpattern of *P* is any substring that is a pattern. Call *P* a < *L*, *W* > pattern (*L* ≤ *W*) if any subpattern of length ≥ *W* has ≥ *L* characters ∈ 𝒜. A pattern *P* has support *K* if it occurs (has instances) in *K* sequences. A pattern *P* can be made more specific by replacing wildcard characters by characters ∈ 𝒜 and/or extending *P* to the left or right. Call *P* maximal with respect to a sequence set *S* if making *P* more specific reduces its total support (irrespective of the number of sequences).

We are now ready to state the behaviour of the algorithm: TEIRESIAS finds all maximal < *L*, *W* > patterns (with support ≥ *K*) in a set *S* of unaligned sequences. In the context of this work, we set *L* = 4, *W* = 16 and *K* = 2. As described below, distances between sequences are calculated using paired residues from patterns. Two residues are said to be paired if they occupy the same column, ie position in different instances of a pattern.

### 3.2 Distance Calculation

The pairwise distance *d^PB^* between two sequences *S_i_* and *S_j_* is computed as follows: first, we filter out patterns with more than one instance in any sequence, for two reasons. a) This reduces the number of false positives, ie patterns with non-homologous instances that arise purely by chance. b) It also ensures that the distance from a sequence to itself is zero, a mathematically desirable property. (This filtration step is meaningful only for patterns, not for words.) Second, all instances of patterns occurring simultaneously in (at least) *S_i_* and *S_j_* are concatenated, resulting in two new sequences *S*′*_ij_* and *S*′*_ji_* of the same length. For example: pattern *P*_1_ = “A.RC” occurs in *S*_1_ and *S*_2_ with instances “AIRC” and “ALRC”, pattern *P*_2_ = “MA..D” occurs in *S*_1_, *S*_2_ and *S*_3_ with instances “MAMAD”, “MAVID” and “MALVD”. After concatenation, we obtain *S*′_12_ = “AIRCMAMAD” and *S*′_21_ = “ALRCMAVID”. Note that a pattern may occur in three or more sequences, in which case we project it on multiple pairs of sequences. For example: *P*_2_ contributes to (*S*′_12_, *S*′_21_), (*S*′_13_, *S*′_31_) and (*S*′_23_, *S*′_32_). Also note that generally *S*′*_ij_* (and *S*′*_ji_*) will differ in length from *S*′*_ik_* (and *S*′*_ki_*) because the patterns occurring simultaneously in (at least) *S_i_* and *S_j_* will differ in number (and number of residues they cover) from those appearing in (at least) *S_k_* and *S_j_*. For example: the length of *S*′_12_ (and *S*′_21_) is 9, whereas the length of *S*′_13_ = “MAMAD” (and *S*′_31_ = “MALVD”) is 5. Third, these new, concatenated sequences are used to estimate pairwise distances. This is done by applying a maximum-likelihood approach that optimizes with respect to a model of amino acid evolution. For the purpose of this work we use the JTT model (Jones et al 1992) as implemented in Protdist from the PHYLIP package (Felsenstein 2005).

Note that the algorithm for calculating distances from patterns is general. Our means for pattern discovery is TEIRESIAS, but, in principle at least, other tools could also be used (eg Apostolico et al 2005). To retain the alignment-free property of our approach, any replacement needs to have that property as well.

### 3.3 Setting parameters

Our rationale for setting TEIRESIAS parameters to *L* = 4, *W* = 16 is as follows. Consider ordinary *k*-mers: higher values for *k* reduce chance occurrences among a set of sequences, thus reducing false positives. We observe that TEIRESIAS patterns and *k*-mers bear a relationship; to this end we introduce *elementary patterns*: an elementary pattern is a < *L*, *W* > pattern with exactly *L* residues. TEIRESIAS discovers maximal < *L*, *W* > patterns using elementary patterns as building blocks during its convolution phase (Rigoutsos and Floratos 1998). For the special case *W* = *L* (no wildcard characters are allowed), setting *L* = *k* leads to elementary patterns capturing a subset of all *k*-mers. The only difference is that *K* = 1 for *k*-mers (a *k*-mer may occur only once) whereas we use *K* = 2 for TEIRESIAS (a pattern must occur in at least two sequences). Thus we see that higher values for *L* reduce the number of false positives. For our distance calculation, however, we need patterns capable of accounting for differences between sequences, hence we require *W* > *L*. Our approach differs from word-based methods: only the use of patterns allows us to formulate and satisfy this requirement. In preliminary experiments on data described in Section 4.2, we tried several higher values for *L* with *W* > *L* first. We found for *L* = 4, *W* = 16 (a ratio of *L/W* = 0.25), the values that we use throughout Section 4, all pairwise distances are defined, ie every pair of sequences is covered by at least one pattern with an instance in both sequences. For *W* = 8, corresponding to a ratio of *L/W* = 0.5, and higher values of *W*, approaching the ratio *L/W* = 0.25, the number of undefined distances is 229, 127, 63, 32, 23, 8, 5, and 2 out of 8667. (On data from Section 4.1, all distances are defined for *W* = 8.)

Undefined distances point towards a problem: some sequence pairs are too divergent—no pair of substrings can be described by (elementary) < *L*, *W* > patterns. The ratio *L/W* determines the minimum similarity any subpattern must possess: it effectively specifies a local similarity threshold. Thus, undefined distances mean that no pair of substrings reach or exceed this threshold. Our solution to the problem is to make sequences more similar by encoding them in a reduced alphabet. Following Rigoutsos et al (2000) and earlier work by Taylor (1986), we choose a reduction based on chemical equivalences: [AG], [DE], [FY], [KR], [ILMV], [QN], [ST], [BZX] where ‘[…]’ groups similar amino acids together, and unlisted amino acids form classes of their own. The phylogenetic distance calculation is based on the original sequence data covered by the resulting patterns; this usually improves phylogenetic accuracy (see Section 4.1.1 and 4.2.2). As a result of encoding sequences, all pairwise distances for eg *L* = 4, *W* = 8 are defined.

We also find that for sufficiently small values of *L*, the phylogenetic accuracy is virtually independent of the particular choice of *L*, and largely depends on the ratio *L/W* (see Appendix). Generally, the accuracy of tree reconstruction improves as the local similarity threshold is lowered, with diminishing improvement and higher computational costs the further it is lowered.

### 3.4 Majority consensus and consistency

One property of TEIRESIAS is that each residue can (and given our parameterization, usually will) participate in multiple patterns. This may lead to situations where a particular residue in one sequence pairs with two or more different residues in a second sequence. For example: assume patterns *P*_1_ and *P*_2_ have overlapping instances in *S*_1_, both covering position *p*_1,19_ = ‘M’. It is possible that their instances in *S*_2_ have two different positions *p*_2,18_ = ‘A’ and *p*_2,23_ = ‘L’ paired to *p*_1,19_. It is not clear how this should be interpreted with respect to homology. We propose a variant, *d^PBMC^* that resolves this conflict by way of (relative) majority consensus and consistency. We discover patterns as before but introduce an intermediate step before distance estimation. We record paired positions across all patterns. For any two sequences *S_i_* and *S_j_*, we accept positions (*p_ik_*, *p_jl_*) if and only if a) *p_ik_* is paired with *p_jn_* more often than with any other position in *S_j_*, b) *p_jl_* is paired with *p_im_* more often than with any other position in *S_i_*, and c) *p_ik_* = *p_im_* and *p_jl_* = *p_jn_*, ie the positions in a) and b) correspond to each other. For example: assume *p*_1,42_ is paired with *p*_2,52_ three times and twice with *p*_2,43_. Hence, condition a) is met. Assume further that *p*_2,52_ is paired with *p*_1,42_ once, meeting condition b). Additionally, condition c) is met, thus we accept pair (*p*_1,42_, *p*_2,52_). This ensures that every residue participates at most once for a given sequence pair in the distance calculation step. For parameters *L* = 4, *W* = 16, the constraints prove to be stringent and discard most of the data.

## 4. Comparison of Alignment-Free Methods

### 4.1 Synthetic Data

We use a birth-death process to model cladogenesis (Nee et al 1994) and sample from several tree distributions. The effects of different taxon sampling strategies are described in Rannala et al (1998). Trees resulting from a birth-death process are rooted, bifurcating and ultrametric; we deviate them from ultrametricity by an additive process to keep the expectation of the phylogenetic distances unchanged.

Using Phylogen V1.1 (Rambaut 2002) we sampled seven sets of 100 four-taxon reference trees each; the parameters were birth = 10.0 and death = 5.0, with extant ∈[40, 133, 400, 133, 4000, 13333, 40000] corresponding to a sample fraction of [0.1, 0.03, …, 0.0001]. The induced pairwise phylogenetic reference distances have medians of [0.71, 1.11, 1.61, 2.08, 2.46, 2.96, 3.39] substitutions per site; their upper and lower quartiles are within 0.35 units of these values. For later use, we label the first, fourth and last set as having small, medium and large phylogenetic distances. Sequences were evolved along the branches of the deviated trees using SEQ-GEN V1.3.2 (Rambaut and Grassly 1997) under the JTT model (Jones et al 1992), and for a sequence length of 1000 amino acids. (Where possible, we parameterized alignment-free methods with the JTT model, or its equilibrium frequencies.)

To compare alignment-free methods with alignment-based methods when the assumption of collinearity is violated, we constructed an additional data set with a wide distribution of phylogenetic distances. We sampled one four-taxon tree each from 100 different distributions specified by sample fractions that varied evenly on a logarithmic scale. The induced pairwise phylogenetic reference distances have a median of 1.77 substitutions per site; the upper and lower quartiles are 2.54 and 1.02, respectively, and the maximum is 4.88. Sequences of length 1000 were evolved as before, and for every sequence set the first and last halves of two sequences were exchanged. This corresponds to a recent domain shuffle event. We deliberately chose an extreme example to show the severity that a non-justified assumption of collinearity can have.

The generated sequences were input to the tested alignment-free methods, and the resulting test distances were used to infer neighbor-joining (NJ: Saitou and Nei 1987) trees. Phylogenetic accuracy is measured by the Robinson-Foulds (RF: Robinson and Foulds 1981) tree metric: we compute the topological difference between a test tree and its corresponding (unrooted) reference tree, and report results for each set. To assess the statistical significance of differences between methods we employ the Friedman test (corrected for tied ranks), followed by Tukey-style posthoc comparisons if a significant difference is found (see eg Siegel and Castellan 1988).

#### 4.1.1 Phylogenetic accuracy

Here, and in Section 4.2.2 we are interested in the accuracy of methods in reconstructing the phylogentic relationships among a set of sequences; we refer to this quantity as phylogenetic accuracy for short. We measure and report the topological differences between test and reference trees: better methods yield fewer differences, and hence have a higher accuracy. When we assess methods based on their ranksums of the Friedman test, better methods obtain lower numbers and rank first.

##### Influence of k and alphabet

Here, we look at the performance of word-based alignment-free methods as a function of the length of *k*-mers and the alphabet in use. We varied *k* from 1 to 9 where possible: the composition distance requires a minimum of *k* = 3. The alphabet consisted of either the original amino acids (AA) or the chemical equivalence classes (CE) from Section 3.3.

For AA sequences, word length *k* = 4 performs best for methods *d^E^*, *d^S^*, *d^F^* and *d^P^* as judged by their ranksums based on phylogenetic accuracy over all seven reference sets. Second- and third-ranking word lengths for *d^E^* and *d^P^* are *k* = 5 and *k* = 3. For *d^F^* these lengths have tied ranks, and for *d^S^* this order is reversed. Method *d^C^* performs best for *k* = 3, with *k* = 4 (*k* = 5) ranking second (third).

For CE sequences, slightly higher values for *k* yield lower ranksums. Methods *d^E^*, *d^F^* and *d^P^* perform best with word length *k* = 5. Second- and third-ranking word lengths for *d^E^* and *d^P^* are *k* = 6 and *k* = 7, for *d^F^* this order is reversed. For *d^S^*, word lengths *k* = 5 and *k* = 6 rank equal best, followed by *k* = 4. Again, method *d^C^* shows a preference for lower values: it performs jointly best for *k* = 4 and *k* = 5, followed by *k* = 3.

**Figure 1:**
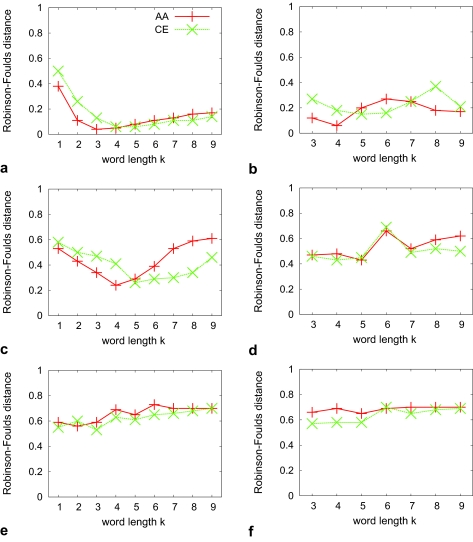
Average Robinson-Foulds distance (Y-axis) for two methods (a,c,e: *d^E^*; b,d,f: *d^C^*) on three reference sets (top to bottom: small, medium and large phylogenetic distances). Each subfigure shows the behaviour as a function of word length *k* (X-axis) under two alphabets (AA: original amino acids, CE: chemical equivalence classes). Points are joined for ease of visual inspection only. The expectation of a tree reconstruction method based on random choice is $\frac{2}{3}$, or about 0.67.

What we have described so far is based on the ranksums over all seven reference sets spanning the relevant space of phylogenetic distances for tree inference. Looking at the phylogenetic accuracy of word-based methods on individual sets with narrow distributions of phylogenetic distances reveals a more complex picture. As expected, the topological difference between test and reference trees increases with increasing phylogenetic reference distances. However, depending on the choice of word length *k*, the absolute values of this difference may vary considerably. This leads to a number of curves with different shapes when plotting the accuracy for a particular method on the Y-axis with the X-axis showing values for *k* (Fig. 1). We find that overall, the choice of method has less impact on the shape of these curves than does phylogenetic distance. Comparing AA with CE sequences shows similarly shaped curves that are shifted to the right for CE. Note that we are interested only in general trends apparent from these curves, and do not attempt to attach significance to differences; this is done below for differences between methods (where word-based methods are parameterized with their best-performing word length).

Figure 1 (a,c,e) shows curves for method *d^E^* for three out of seven different reference sets with small, medium and large phylogenetic distances. The curves for methods *d^S^*, *d^F^* and *d^P^* are similar and appear in the appendix. The curve for medium distances corresponds to our overall findings. Small and large distances hint at a better performance for small values of *k*. Inspection of these plots for the remaining methods fails to identify a single best *k*. Figure 1 (b,d,f) for *d^C^* reveals some striking peculiarities of this method. In these three sets, for *k* = 6 the topological difference between test and reference trees is often the highest, even though the neighbor value *k* = 5 may yield a low topological difference (medium distances): thus the parameter space is uneven, more so than for other methods.

Taken together, these results indicate that, depending on the phylogenetic distance of the sequences, the word length most appropriate for analysis of a particular data set may well be different from the one performing best over all sets tested here. In practice, intermediate word lengths (performing best overall in our setup) should work well on a large variety of data sets.

##### Statistical significance

Here, we conduct a comprehensive comparison of all methods: we show their phylogenetic accuracy and assign statistical significance to our findings. For *k*-mer-based methods, we select the best-performing word lengths on the two alphabets as described above. We compare these methods to *d^ACS^*, *d^LZ^* and *d^W^*, and to two variants of the pattern-based approach: *d^PB^* and *d^PBMC^*. As a baseline, we include *d^ML^*, the maximum-likelihood (ML) estimate of phylogenetic distances between the (already correctly aligned) sequences.

Table 1 lists selected methods in ranksum order based on all 700 trees, from best to worst. For every method, we show the number of incorrectly reconstructed trees in each of the seven sets. Recall that unrooted, bifurcating four-taxon trees can be reconstructed either correctly or incorrectly: the RF distance will be 0 or 1, with no intermediate values possible. There are 3 distinct possible topologies for such trees, hence we expect a method based on random choice to correctly reconstruct $\frac{1}{3}$ of all trees, and conversely, to incorrectly reconstruct $\frac{2}{3}$ of all trees. For each reference set, the expected RF distance of the random tree reconstruction method thus amounts to about 67 out of 100 trees.

The test statistic of the Friedman test (corrected for tied ranks) is *F_R_* = 709.6 (*N* = 700, *k* = 19). This is highly significant (*p*-value < 10^−10^) at or beyond the *α* = 0.05 level. Significant differences are found between the following pairs (numbers refer to entries in column ‘#’ of Table 1): method 1 *vs* methods 19–4, method 2 *vs* methods 19–5, method 3 and 4 *vs* methods 19–15, and methods 5–16 *vs* method 19. Thus the performance of most alignment-free methods as tested here is statistically indistinguishable from one another. The ranksums of methods 5–14 range from 6951.0 to 7074.5, differing by ≤ 123.5. However, the pattern-based method *d^PB^* with CE, *L* = 4, *W* = 16 (ranksum: 6058.0) is significantly better than all alignment-free methods not based on patterns. The ML estimate based on the correct alignment, *d^ML^*, is significantly better than all traditional alignment-free methods and the pattern-based method working on original AA sequences. Note that *d^ML^* is not significantly better than the two best-performing pattern-based variants working on CE sequences.

By far the worst method tested here is the W-metric *d^W^* (ranksum: 8281.0): differences to nearly all other methods are significant. The lack of phylogenetic accuracy originates from being based on 1-mers. For comparison, *d^E^* with AA, *k* = 1 incorrectly reconstructs the following number of trees for the seven reference sets: 38, 30, 39, 53, 58, 66, 59. These numbers are quite similar to the ones in Table 1, as are the numbers for equally parameterized methods *d^S^* and *d^F^*. In the case of *d^P^*, however, they are 59, 56, 65, 75, 59, 71, 65. This is an artifact of the method for *k* = 1 (and to some extent for *k* = 2) and vanishes for higher values. Also apparent is the poor performance of both parameterizations of *d^LZ^* and *d^C^*, the Lempel-Ziv and composition distances, respectively, with ranksums between 7359.5 and 7635.0.

##### Domain shuffling

We now describe our findings from the reference set with simulated domain shuffling data. We apply the same alignment-free methods with parameter settings as before on the unaligned, partly shuffled sequences. Additionally, we run a number of multiple sequence alignment (MSA) programs on these data (Thompson et al 1994; Morgenstern 1999; Edgar 2004b; Do et al 2005), and estimate ML distances from these alignments (*d*^ClustalW^, *d*^Dialign^, *d*^MUSCLE^ and *d*^ProbCons^, respectively). Aligning sequences that contain shuffled domains is inappropriate and our setup corresponds to an undesirable situation where, for example, in an automated environment tests have failed to detect the presence of domain shuffling. Hence, distances are estimated from alignments where not all homologous residues can possibly be aligned, and it is likely that in fact a substantial fraction of aligned residues are non-homologous.

The Friedman test (*F_R_* = 270.3, *N* = 100, *k* = 22) detects the presence of a difference that is highly significant (*p*-value < 10 ^−10^) at or beyond the *α* = 0.05 level. However, pairwise differences are statistically significant only between *d*^ClustalW^ and all other methods; this is likely due to lack of statistical power. Two parameterizations (CE and AA) of the pattern-based method *d^PB^*, *L* = 4, *W* = 16, rank jointly first with ranksums of 1011.5: they reconstruct 11 out of 100 trees incorrectly. This is followed jointly by *d^PBMC^*, with CE, *L* = 4, *W* = 16, and *d^S^* and *d^F^*, both with CE, *k* = 5. Their ranksums are 1066.5, and reconstruct 16 incorrect trees. The number of incorrectly reconstructed trees for alignment-based approaches are as follows (ranksum in parentheses): *d*^ClustalW^: 71 (1671.5), *d*^MUSCLE^: 28 (1198.5), *d*^ProbCons^: 25 (1165.5), and *d*^Dialign^:21 (1121.5). Three out of four alignment-based approaches are among the seven worst-ranking methods. Interestingly *d*^Dialign^, the best-performing of these approaches, uses a local alignment strategy; it occupies rank eleven jointly with two other methods. Conversely, what we just described means that eg *d^LZ^*, working on AA sequences, one of the worst-performing alignment-free methods as tested here, has a higher phylogenetic accuracy than three out of four combinations of MSA program and ML estimate, and even *d^W^* is significantly better than *d*^ClustalW^ on these data. Overall, the results show that alignment-free methods may perform better than alignment-based approaches, especially on non-collinear sequence data, as alignment-free methods do not make assumptions of collinearity.

### 4.2 Empirical data

We use the data from version 2 of the original BAliBASE sets (Thompson et al 1999). They consist of 141 manually curated benchmark alignments that are organized in five reference sets. Their purpose is to support tests of alignment tools under a variety of conditions: Set 1 is made up of roughly equidistant sequences that are divided into nine subsets according to their sequence conservation and alignment length. Set 2 contains sequence families that are aligned with a highly divergent orphan sequence. Set 3 aligns subgroups with less than 25 percent identity between them. Set 4 consists of sequences with N- or C-terminal extensions, ie the sequences are not trimmed at alignment boundaries. Set 5 is complementary to set 4: some sequences contain internal insertions. Two alignments contain only three sequences each and are not considered for evaluation of phylogenetic accuracy, as there is only one corresponding unrooted tree topology. The remaining 139 alignments consist of between 4 and 28 sequences each.

For each reference alignment, we estimate phylogenetic reference distances using Protdist, and reconstruct both neighbor-joining (NJ) and Fitch-Margoliash (FM: Fitch and Margoliash 1967) reference trees. The topological difference between a test tree and its corresponding reference tree is measured by the Robinson-Foulds (RF) and the Quartet (Q) distance (Estabrook et al 1985; implemented in QDist: Mailund and Pedersen 2004). As these are empirical data, we cannot know the true tree along which the sequences evolved; however, we find that by using a large number of trees, and four combinations of tree reconstruction method and tree topology metric (RF-NJ, RF-FM, Q-NJ, Q-FM), we are able to rank methods robustly. Statistical significance is assessed as in Section 4.1.

#### 4.2.1 Phylogenetic distances

**Figure 2:**
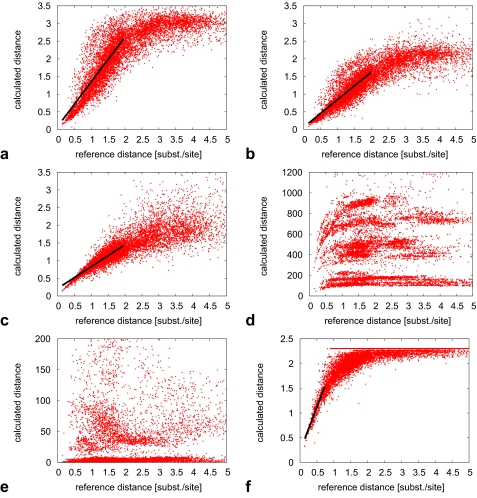

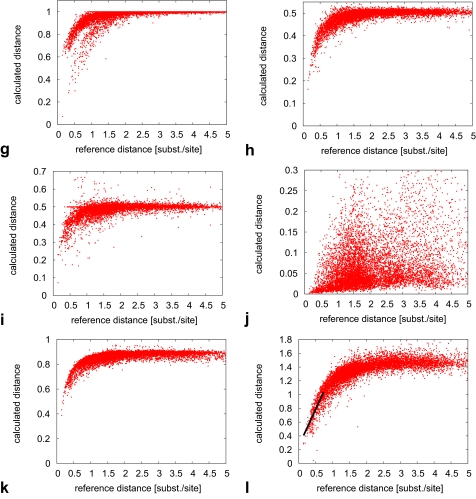
Pairwise phylogenetic reference distances (X-axis) plotted against corresponding calculated distances (Y-axis). Methods and parameters are as follows: a) *d^PB^* with *L* = 4, *W* = 16, CE, b) *d^PB^* with *L* = 4, *W* = 16, AA, c) *d^PBMC^* with *L* = 4, *W* = 16, CE, d) *d^E^* with *k* = 4, AA, e) *d^S^* with *k* = 4, AA, f) *d^F^* with *k* = 4, AA, g) *d^P^* with *k* = 4, AA, h) *d^C^* with *k* = 3, AA, i) *d^C^* with *k* = 5, CE, j) *d^W^*, k) *d^LZ^* with AA, l) *d^ACS^* with A A.

Here, we inspect the behaviour of pairwise phylogenetic distances. The BAliBASE alignments yield 8667 reference distances, of which < 2.5% have ≥ 5.0 substitutions per site (36 distances are ≥ 10.0). In what follows, we consider reference distances < 5.0, ie < 500 PAMs. Note that “distances of 250–300 PAM units are commonly considered as the maximum for reasonable distance estimation” (Sonnhammer and Hollich 2005). Figure 2 contains scatterplots where the X-axis refers to the aforementioned reference distances. The Y-axis shows the corresponding phylogenetic distances obtained using selected alignment-free methods with original AA sequences (and, if the methods are word-based, values for *k* as in Table 1). Additionally, we show distances obtained from CE sequences where the distribution differs noticably.

Ideally, a method calculates distances that are linear to the reference distances, as this allows for a simple (linear) transformation of its results to obtain the actual values (which are of intrinsic interest). We find that no method is linear over the whole range considered. Therefore, we apply linear regression and report results where the linear range is substantial. This is somewhat subjective; however, we note that it serves as a descriptive tool to understand the accuracy of the methods.

Figure 2a for *d^PB^* with CE sequences, *L* = 4, *W* = 16, shows a linear relationship between reference and estimated distances for up to 2.0–2.5 substitutions per site. Linear regression for all points below 2.0 yields *y* = 0.0794 + 1.2698*x* with a correlation coefficient (CC) of 0.8015. Higher distances are increasingly underestimated as saturation comes into effect and limits most distances (> 99%) to values < 3.5.

When patterns are discovered using the original BAliBASE sequences (Fig. 2b) as opposed to using CE, the resulting distances are approximated by a line with a lesser slope (*y* = 0.0668 + 0.7790*x*, CC = 0.7970); saturation limits most distances (> 99%) to values < 2.5. For the variant *d^PBMC^* with CE sequences, *L* = 4, *W* = 16 (Fig. 2c), the linear relationship extends at least up to 2.0–2.5 substitions per site and the slope is roughly half that of the equally parameterized *d^PB^*(*y* = 0.2108 + 0.6201*x*, CC = 0.8641). Again, most distances (> 99%) are limited to values < 3.5, however this curve shows less saturation and more scatter.

The (squared) Euclidean distance *d^E^* does not yield values that can be interpreted in units of substitutions per site. Instead, they relate to mismatch counts (Blaisdell 1989) and are therefore sequence length-dependent. Correspondingly, the distances do not show a single discernable linear relationship (Fig. 2d for *k* = 4, with AA sequences). Most of the data (> 99%) have an Euclidean distance of < 1200; data for *k* = 5, with CE sequences are very similar and omitted here.

Similarly, *d^S^* has no single discernable linear relationship, with most data (> 99%) taking on numerical values of < 200 (*k* = 4, AA, Fig. 2e). However, parameters *k* = 5, CE yield distances with most values (> 99%) being < 4000, although the scatterplots look almost identical.

We find a linear relationship between reference and *d^F^* distances for up to about 0.75 substitutions per site (Fig. 2f). Linear regression for all points below this cutoff yields *y* = 0.2304 + 1.7823*x*, CC = 0.8558. We find 25.5% of all pairwise distances are − log(*ɛ*), ie would be undefined without adding *ɛ* as no *k*-mer is common to a sequence pair. This problem occurs especially for short, divergent sequences in set 1 of BAliBASE. For *k* = 5, with CE sequences, this number drops down considerably to 15.1%, and the plot exhibits more scatter.

A similar problem occurs for *d^P^* (Fig. 2g), which is also based on common *k*-mers. Here 30.8% of all pairwise distances yield the value 1.0; this higher percentage is possibly caused by numerical instabilities when multiplying small probabilities. As before, parameterization with *k* = 5, CE reduces the percentage considerably, to 21.0%, and increases scatter.

The scatterplots for the composition distance *d^C^* differ between parameterization *k* = 3, AA and *k* = 5, CE (Fig. 2h,i). The former version exhibits somewhat linear behaviour, with no value ≥ 0.55, whereas the latter version shows no such linear behaviour and is limited to about 0.75. More importantly, in this latter version 13.1% of its distances are exactly 0.5 (within the usual precision limits imposed by implementations of floating point numbers), and 30.2% are ∈ [0.4999, 0.5001]. For the former version, just 6 distances amount to 0.5, and 60 are ∈ [0.4999, 0.5001].

We find no discernable relationship between reference distances > 0.5 and distances produced by the W-metric (Fig. 2j). This likely explains why it turns out to be the worst of all tested methods here. The distances are mostly (> 99%) limited to values < 0.3.

The two distributions of values for parameterizations of the Lempel-Ziv distance *d^LZ^* with both AA (Fig. 2k) and CE sequences are very similar, with CE showing more scatter.

The method *d^ACS^* shows a linear relationship between reference and calculated distances for up to about 0.75–1.0 substitutions per site (Fig. 2l). Linear regression for all points below 0.75 yields *y* = 0.2551 + 1.0643*x* (CC = 0.8063). We find most distances > 99% are limited to values < 1.65. For CE sequences, most distances > 99% are limited to values < 1.1.

Comparing the various distributions, we find that all three versions of our pattern-based approach yield pairwise distances that exhibit a linear relationship to phylogenetic reference distances for up to about 2.0–2.5 substitutions per site. This constitutes a considerable increase from a maximum of 0.75–1.0 substitutions per site for methods *d^ACS^* and *d^F^*. The linear relationship is a desirable property, and likely explains the higher phylogenetic accuracy of the pattern-based approach.

#### 4.2.2 Phylogenetic accuracy

##### Statistical significance

Table 2 lists selected alignment-free methods and four approaches based on the ML distance estimate from automated alignments. To obtain combined ranksums for statistical analysis, we average the normalized topological differences over all four combinations of tree reconstruction method and tree distance measure (RF-NJ, RF-FM, Q-NJ, Q-FM) based on 139 sequence sets. The combined ranksums range from 975.5 to 2338.0. This is a slightly wider range than for each individual combination, for which the extreme values are ∈ [987.5, 1058.5] and ∈ [2280.0, 2312.0]. The first and last six methods are ranked identically between the combined analysis and combination RF-NJ. The average normalized topological differences for this combination are shown in Table 2 for all five BAliBASE reference sets.

The Friedman test statistic *F_R_* = 579.2 (*N* = 139, *k* = 22) is highly significant (*p*-value < 10 ^−10^) at or beyond the *α* = 0.05 level. Significant differences are found between the following pairs (numbers refer to column ‘#’ of Table 2): methods 1–3 *vs* methods 22–6, method 4 *vs* methods 22–9, method 5 *vs* methods 22–12, methods 6 *vs* methods 22–20, methods 7–18 *vs* methods 22 and 21, and method 19 *vs* method 22. This implies that all alignment-based approaches yield significantly better results than any of the alignment-free methods not based on patterns, except for *d*^Dialign^ *vs d^ACS^* with CE. Additionally, three out of four alignment-based approaches (ranksums: 975.5–1008.0) are significantly better-performing than two pattern-based variants although not than *d^PB^* with CE, *L* = 4, *W* = 16 (ranksum: 1239.0). This version significantly outperforms all but four alignment-free methods not based on patterns. Again, both parameterizations of the composition distance and the W-metric trail behind, with ranksums of 1968.5, 2171.0 and 2338.0, respectively. Similarly to our previous analysis, most alignment-free methods are statistically indistinguishable. Ranksums for methods 8–18 range from 1583.0 to 1755.0, a difference of 172.0. On this data set, *d^PBMC^* is only marginally better (ranksum: 1570.0). A possible explanation is apparent from Table 1. There, *d^PBMC^* performs poorly on reference set 7 (large phylogenetic distances) in comparison to both parameterizations of *d^PB^*. We find 1986 out of 8667 pairwise phylogenetic reference distances (ie 22.9%) in BAliBASE have ≥ 3.0 substitutions per site.

## 5. Conclusions

We present here for the first time a comprehensive evaluation of alignment-free methods with respect to their accuracy in reconstructing the phylogenetic relationship among a set of sequences. We show that the performance of most methods is statistically indistinguishable from another. The pattern-based approach as introduced by us here proved to be significantly better than most previously established methods. At the same time, we provide a point of reference for alignment-free methods by measuring the maximum-likelihood (ML) distance estimate based on reference and automated alignments. In our tests, we found the best-performing version of our pattern-based approach *d^PB^* to be statistically indistinguishable from this estimation, while most alignment-free methods rank significantly worse on ordinary, non-shuffled sequences. However, on non-collinear sequences we show that most alignment-free methods reconstruct trees more accurately than approaches based on automated alignments. In fact, these alignments should not be used as they largely align non-homologous residues. The inspection of ClustalW alignments reveals artifacts of this method: it forces most residues to align with other (non-homologous) residues, and places too few gaps.

In all three experiments we found that *d^PB^* ranks higher than the equally parameterized variant *d^PBMC^*, although not significantly. The latter variant intuitively seems to do more justice to the concept of homology; however, we cannot provide a satisfying explanation for its worse perfomance. All three versions of our pattern-based approach result in distances that show a linear relationship to phylogenetic reference distances over a substantially longer range than any other alignment-free method considered here.

We also utilized a different alphabet for amino acid (AA) sequences based on chemical equivalences (CE). We found that *d^PB^* with CE yields results as good as *d^PB^* with AA, and often yields considerably increased phylogenetic accuracy. We also tested the other alignment-free methods on sequences encoded in this alphabet. For any given parameterization, CE always improves performance on set 7 (large phylogenetic distances: *cf* Table 1, and also Fig. 1 e,f) by 4 to 12 (out of 100) fewer incorrectly reconstructed trees. This probably explains why methods parameterized with CE *vs* AA perform better on BAliBASE than on the synthetic dataset. Note that we did not try to optimize the alphabet; certainly, there are many different choices (see eg Edgar 2004a). Also, our findings seem to contradict results of that study. Edgar (2004a) found that *k*-mers based on various compressed alphabets did not improve the correlation coefficient between *d^F^* and percent identity as compared to using the original alphabet. In our own experiments we found the correlation coefficient between estimated and reference distance to be a bad estimator of phylogenetic accuracy (data not shown).

Finally, based on the data in Table 1, we note that there is ample room for further improvement of alignment-free methods: compare the results for *d^ML^* with *d^PB^*, especially on reference sets 5 to 7, ie large phylogenetic distances. Quite likely this will be possible only if new alignment-free methods incorporate models of sequence change.

## Figures and Tables

**Figure 3:**
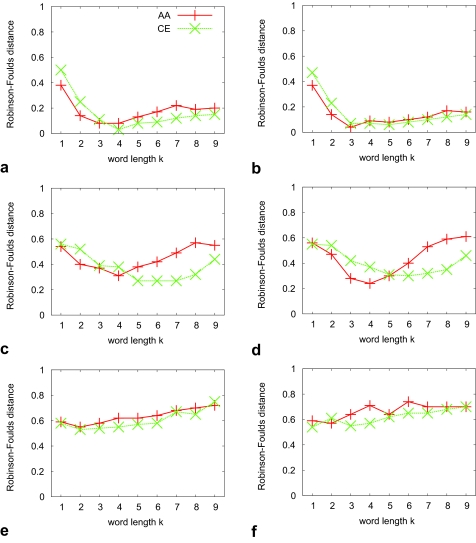
Average Robinson-Foulds distance (Y-axis) for two methods (a,c,e: *d^S^*; b,d,f: *d^F^*) on three reference sets (top to bottom: small, medium and large phylogenetic distances). Each subfigure shows the behaviour as a function of word length *k* (X-axis) under two alphabets (AA: original amino acids, CE: chemical equivalence classes). Points are joined for ease of visual inspection only. The expectation of a tree reconstruction method based on random choice is $\frac{2}{3}$, or about 0.67.

**Figure 4:**
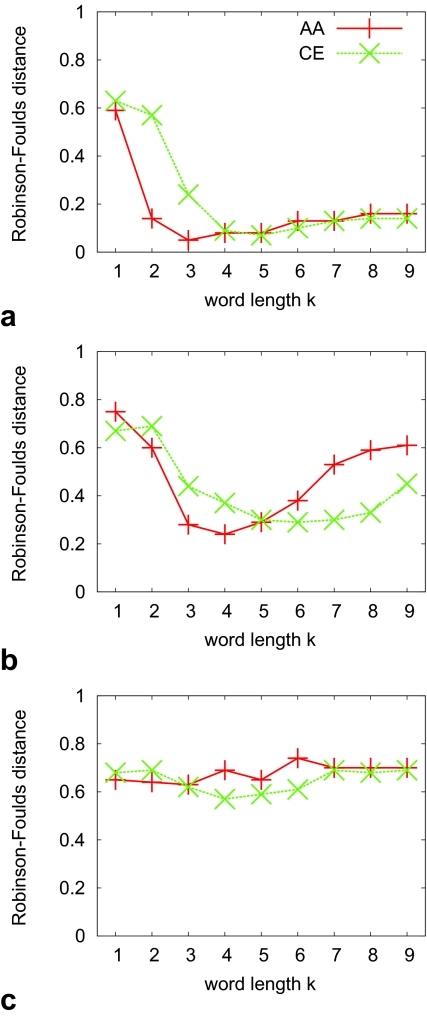
Average Robinson-Foulds distance (Y-axis) for method *d^P^* on three reference sets (top to bottom: small, medium and large phylogenetic distances). Each subfigure shows the behaviour as a function of word length *k* (X-axis) under two alphabets (AA: original amino acids, CE: chemical equivalence classes). Points are joined for ease of visual inspection only. The expectation of a tree reconstruction method based on random choice is $\frac{2}{3}$, or about 0.67.

**Table 1:** Shown are the number of incorrectly reconstructed trees (out of 100) for each synthetic reference set and method. The order is based on ranksums ∑*_R_*; for each word-based method (and alphabet 𝒜), we include the best-performing word length *k* (method *d^W^* can only take on a value of 1). The expectation of a tree reconstruction method based on random choice is about 67.

					Synthetic reference set						
#	∑_R_	Method	𝒜	k	1	2	3	4	5	6	7
1	5640.0	*d^ML^*	AA	–	2	2	7	12	13	18	17
2	6058.0	*d^PB^*	CE	–	3	2	10	9	19	29	43
3	6390.5	*d^PBMC^*	CE	–	3	3	10	10	23	42	59
4	6523.5	*d^PB^*	AA	–	4	2	7	18	38	45	50
5	6951.0	*d^S^*	CE	5	8	6	19	27	45	47	57
6	6960.5	*d^P^*	AA	4	8	1	14	24	44	50	69
7	6970.0	*d^E^*	AA	4	5	2	17	24	46	48	69
8	6989.0	*d^ACS^*	AA	–	9	3	17	24	49	52	59
9	6998.5	*d^P^*	CE	5	7	4	18	30	47	49	59
10	7036.5	*d^S^*	AA	4	8	8	20	31	43	46	62
11	7036.5	*d^F^*	AA	4	9	2	14	24	48	50	71
12	7055.5	*d^E^*	CE	5	6	7	21	26	51	48	61
13	7065.0	*d^ACS^*	CE	–	10	7	21	30	43	55	55
14	7074.5	*d^F^*	CE	5	6	6	21	31	47	49	62
15	7359.5	*d^LZ^*	CE	–	6	5	28	39	49	66	59
16	7378.5	*d^LZ^*	AA	–	3	8	21	34	53	64	71
17	7597.0	*d^C^*	AA	3	12	14	31	47	49	58	66
18	7635.0	*d^C^*	CE	5	15	14	32	45	54	63	58
19	8281.0	*d^W^*	AA	(1)	43	34	35	55	50	71	61

**Table 2:** Shown are average topological differences for each BAliBASE reference set and method; these average values are based on neighbor-joining trees and the normalized Robinson-Foulds measure. The order is based on combined ranksums ∑*_R_* (see text for details); parameters for word-based methods are as in Table 1.

					BAliBASE reference set				
#	∑***R***	Method	𝒜	k	1	2	3	4	5
1	975.5	*d*^MUSCLE^	AA	–	0.240	0.370	0.274	0.442	0.244
2	1005.0	*d*^ClustalW^	AA	–	0.210	0.389	0.337	0.423	0.249
3	1008.0	*d*^ProbCons^	AA	–	0.204	0.396	0.336	0.474	0.164
4	1190.5	*d*^Dialign^	AA	–	0.310	0.428	0.399	0.646	0.270
5	1239.0	*d^PB^*	CE	–	0.306	0.510	0.357	0.478	0.330
6	1453.0	*d^PB^*	AA	–	0.404	0.563	0.398	0.524	0.428
7	1570.0	*d^PBMC^*	CE	–	0.440	0.557	0.428	0.609	0.460
8	1583.0	*d^ACS^*	CE	–	0.394	0.583	0.433	0.591	0.366
9	1603.0	*d^P^*	CE	5	0.408	0.570	0.442	0.568	0.412
10	1625.5	*d^LZ^*	AA	–	0.431	0.569	0.389	0.642	0.511
11	1632.5	*d^E^*	CE	5	0.408	0.593	0.464	0.570	0.410
12	1646.0	*d^F^*	CE	5	0.396	0.575	0.467	0.569	0.400
13	1703.0	*d^ACS^*	AA	–	0.483	0.579	0.401	0.660	0.451
14	1705.0	*d^E^*	AA	4	0.508	0.578	0.418	0.622	0.489
15	1706.5	*d^LZ^*	CE	–	0.421	0.622	0.440	0.628	0.437
16	1707.5	*d^P^*	AA	4	0.496	0.589	0.419	0.637	0.469
17	1751.5	*d^F^*	AA	4	0.515	0.580	0.431	0.666	0.475
18	1755.0	*d^S^*	CE	5	0.446	0.636	0.491	0.603	0.375
19	1830.0	*d^S^*	AA	4	0.513	0.624	0.450	0.607	0.528
20	1968.5	*d^C^*	AA	3	0.481	0.681	0.525	0.570	0.588
21	2171.0	*d^C^*	CE	5	0.535	0.776	0.642	0.796	0.611
22	2338.0	*d^W^*	AA	(1)	0.585	0.885	0.795	0.897	0.720

**Table 3:** Shown are the number of incorrectly reconstructed trees (out of 100) for synthetic reference set 4 (medium phylogenetic distances). Method *d^PB^* was run using both alphabets 𝒜 and various values for *L* and *W*: a) *L* = 4, *W* ∈ [8, 12, 16]; b) *L* = 3, *W* ∈ [6, 9, 12]; c) *L* = 2, *W* ∈ [4, 6, 8].

		ratio L/W		
𝒜	L	0.50	0.33	0.25
CE	4	14	13	9
CE	3	15	12	12
CE	2	16	11	9
AA	4	36	26	18
AA	3	27	16	15
AA	2	25	13	13

## Appendix

### Setting parameters

Table 3 exemplifies that the accuracy of tree reconstruction for the pattern-based approach depends largely on the ratio of values for parameters *L* and *W* for a given alphabet: the number of incorrectly reconstructed trees decreases as the ratio gets smaller. (We note that *L* = 2, *W* = 3 are the smallest usable values to discover patterns that contain wildcard characters; such patterns are required for calculating distances.) It is also apparent that *L* = 4 benefits from alphabet CE (as compared to AA) much more strongly than does *L* = 2. This effect increases with increasing phylogenetic distance between sequences.

### Influence of k and alphabet

Figures 3 and 4 are similar to Figure 1 and show curves for word-based methods *d**^S^*, *d**^F^* and *d**^P^*. Note how the shape of the curves varies little between methods and is mostly dependent on phylogenetic distance. Figure 4 also visualises the extent to which *d**^P^* is worse than other word-based methods for word length *k* =1 and, to some degree, for *k* = 2.

## References

[b1-ebo-02-359] Apostolico A, Comin M, Parida L (2005). Conservative extraction of overrepresented extensible motifs. In.

[b2-ebo-02-359] Blaisdell B (1986). A measure of the similarity of sets of sequences not requiring sequence alignment.. Proc. Natl Acad. Sci. U. S. A.

[b3-ebo-02-359] Blaisdell B (1989). Average values of a dissimilarity measure not requiring sequence alignment are twice the averages of conventional mismatch counts requiring sequence alignment for a computer-generated model system.. J. Mol. Evol.

[b4-ebo-02-359] Burstein D, Ulitsky I, Tuller T, Chor B (2005). Information theoretic approaches to whole genome phylogenies. In.

[b5-ebo-02-359] Do C, Mahabhashyam M, Brudno M, Batzoglou S (2005). ProbCons: probabilistic consistency-based multiple sequence alignment.. Genome Res.

[b6-ebo-02-359] Edgar R (2004a). Local homology recognition and distance measures in linear time using compressed amino acid alphabets. Bioinformatics.

[b7-ebo-02-359] Edgar R (2004b). MUSCLE: multiple sequence alignment with high accuracy and high throughput.. Nucleic Acids Res.

[b8-ebo-02-359] Estabrook G, McMorris F, Meacham C (1985). Comparison of undirected phylogenetic trees based on subtrees of four evolutionary units.. Syst. Zool.

[b9-ebo-02-359] Felsenstein J (2005). PHYLIP (phylogeny inference package) version 3.65.

[b10-ebo-02-359] Fitch W, Margoliash E (1967). Construction of phylogenetic trees. Science.

[b11-ebo-02-359] Gentleman J, Mullin R (1989). The distribution of the frequency of occurrence of nucleotide subsequences, based on their overlap capability. Biometrics.

[b12-ebo-02-359] Hao B, Qi J (2004). Prokaryote phylogeny without sequence alignment: from avoidance signature to composition distance.. J., Bioinf. and Computat. Biol.

[b13-ebo-02-359] Henikoff S, Henikoff J (1992). Amino acid substitution matrices from protein blocks.. Proc. Natl Acad. Sci. U. S. A.

[b14-ebo-02-359] Jones D, Taylor W, Thornton J (1992). The rapid generation of mutation data matrices from protein sequences.. Comput. Appl. Biosci.

[b15-ebo-02-359] Lempel A, Ziv J (1976). On the complexity of finite sequences. IEEE Trans. Inform. Theory.

[b16-ebo-02-359] Mailund T, Pedersen C (2004). Qdist—quartet distance between evolutionary trees. Bioinformatics.

[b17-ebo-02-359] Morgenstern B (1999). DIALIGN 2: improvement of the segment-to-segment approach to multiple sequence alignment. Bioinformatics.

[b18-ebo-02-359] Nee S, May R, Harvey P (1994). The reconstructed evolutionary process.. Phil. Trans. R. Soc. B.

[b19-ebo-02-359] Otu H, Sayood K (2003). A new sequence distance measure for phylogenetic tree reconstruction. Bioinformatics.

[b20-ebo-02-359] Pham T, Zuegg J (2004). A probabilistic measure for alignment-free sequence comparison. Bioinformatics.

[b21-ebo-02-359] Rambaut A (2002). PhyloGen: phylogenetic tree simulator package.

[b22-ebo-02-359] Rambaut A, Grassly N (1997). Sequence-Generator: an application for the Monte Carlo simulation of molecular sequence evolution along phylogenetic trees.. Comput. Appl. Biosci.

[b23-ebo-02-359] Rannala B, Huelsenbeck J, Yang Y, Nielsen R (1998). Taxon sampling and the accuracy of large phylogenies.. Syst. Biol.

[b24-ebo-02-359] Rigoutsos I, Floratos A (1998). Combinatorial pattern discovery in biological sequences: the TEIRESIAS algorithm. Bioinformatics.

[b25-ebo-02-359] Rigoutsos I, Floratos A, Parida L, Gao Y, Platt D, Huynh T (2000, 10). TEIRESIAS: a vade mecum.

[b26-ebo-02-359] Robinson DF, Foulds LR (1981). Comparison of phylogenetic trees.. Math. Biosci.

[b27-ebo-02-359] Saitou N, Nei M (1987). The neighbor-joining method: a new method for reconstructing phylogenetic trees.. Mol. Biol. Evol.

[b28-ebo-02-359] Siegel S, Castellan N (1988). Nonparametric statistics for the behavioral sciences (2nd ed) Boston, Massachusetts:.

[b29-ebo-02-359] Snel B, Huynen M, Dutilh B (2005). Genome trees and the nature of genome evolution.. Annu. Rev. Microbiol.

[b30-ebo-02-359] Sonnhammer E, Hollich V (2005). *Scoredist*: a simple and robust protein sequence distance estimator. BMC Bioinformatics.

[b31-ebo-02-359] Taylor W (1986). The classification of amino acid conservation.. J. Theor. Biol.

[b32-ebo-02-359] Thompson J, Higgins D, Gibson T (1994). CLUSTAL W: improving the sensitiv ity of progressive multiple sequence alignment through sequence weighting, position specific gap penalties and weight matrix choice.. Nucleic Acids Res.

[b33-ebo-02-359] Thompson J, Plewniak F, Poch O (1999). BAliBASE: a benchmark alignment database for the evaluation of multiple alignment programs. Bioinformatics.

[b34-ebo-02-359] Van Helden J (2004). Metrics for comparing regulatory sequences on the basis of pattern counts. Bioinformatics.

[b35-ebo-02-359] Vinga S, Almeida J (2003). Alignment-free sequence comparison—a review. Bioinformatics.

[b36-ebo-02-359] Vinga S, Gouveia-Oliveira R, Almeida J (2004). Comparative evaluation of word composition distances for the recognition of SCOP relationships. Bioinformatics.

[b37-ebo-02-359] Wu T-J, Burke J, Davison D (1997). A measure of DNA sequence dissimilarity based on the Mahalanobis distance between frequencies of words. Biometrics.

[b38-ebo-02-359] Wu TJ, Huang Y-H, Li L-A (2005). Optimal word sizes for dissimilarity measures and estimation of the degree of dissimilarity between DNA sequences. Bioinformatics.

